# Fine-tuning neural excitation/inhibition for tailored ketamine use in treatment-resistant depression

**DOI:** 10.1038/s41398-021-01442-3

**Published:** 2021-05-29

**Authors:** Erik D. Fagerholm, Robert Leech, Steven Williams, Carlos A. Zarate, Rosalyn J. Moran, Jessica R. Gilbert

**Affiliations:** 1 Department of Neuroimaging, King’s College London, London, UK; 2 Experimental Therapeutics and Pathophysiology Branch, NIMH, NIH, Bethesda, MD USA

**Keywords:** Depression, Neuroscience

## Abstract

The glutamatergic modulator ketamine has been shown to rapidly reduce depressive symptoms in patients with treatment-resistant major depressive disorder (TRD). Although its mechanisms of action are not fully understood, changes in cortical excitation/inhibition (E/I) following ketamine administration are well documented in animal models and could represent a potential biomarker of treatment response. Here, we analyse neuromagnetic virtual electrode time series collected from the primary somatosensory cortex in 18 unmedicated patients with TRD and in an equal number of age-matched healthy controls during a somatosensory ‘airpuff’ stimulation task. These two groups were scanned as part of a clinical trial of ketamine efficacy under three conditions: (a) baseline; (b) 6–9 h following subanesthetic ketamine infusion; and (c) 6–9 h following placebo-saline infusion. We obtained estimates of E/I interaction strengths by using dynamic causal modelling (DCM) on the time series, thereby allowing us to pinpoint, under each scanning condition, where each subject’s dynamics lie within the Poincaré diagram—as defined in dynamical systems theory. We demonstrate that the Poincaré diagram offers classification capability for TRD patients, in that the further the patients’ coordinates were shifted (by virtue of ketamine) toward the stable (top-left) quadrant of the Poincaré diagram, the more their depressive symptoms improved. The same relationship was not observed by virtue of a placebo effect—thereby verifying the drug-specific nature of the results. We show that the shift in neural dynamics required for symptom improvement necessitates an increase in both excitatory and inhibitory coupling. We present accompanying MATLAB code made available in a public repository, thereby allowing for future studies to assess individually tailored treatments of TRD.

## Introduction

Ketamine’s rapid antidepressant efficacy was first demonstrated in a clinical trial by Berman et al.^1^—a study that led to an intense focus on the glutamatergic system’s putative role in mood disorders, including major depressive disorder (MDD)^2,3^ and bipolar depression^4,5^. While still elusive, a mechanistic explanation for ketamine’s antidepressant effects could lead to the development of novel, rapid-acting therapeutics or adjunctive treatment options better tailored to individual patients, without unwanted psychoactive side effects and abuse potential. Ketamine, a non-competitive N-methyl-d-aspartate (NMDA) receptor antagonist^6^, is thought to exert its anaesthetic, dissociative, and psychotomimetic actions via NMDA receptor inhibition^7^. However, multiple direct and indirect mechanisms resulting from NMDA receptor inhibition that culminate in increased brain-derived neurotrophic factor (BDNF) release^8^, and α-amino-3-hydroxy-5-methyl-4-isoxazolepropionic acid (AMPA) surface expression^9,10^ have been posited to contribute to its antidepressant actions^11^.

NMDA receptor blockade by subanesthetic dose ketamine of fast-spiking gamma-aminobutyric acid (GABA)-ergic interneurons, particularly the parvalbumin basket cells, leads to local inhibition of interneuron tonic firing and subsequent disinhibition of pyramidal neurons downstream of NMDA receptor antagonism^12,13^. This produces an immediate glutamate surge^14^, activating the mammalian target of rapamycin complex 1 (mTORC1) pathway^15^, increasing BDNF release, and activating downstream signalling pathways that stimulate synapse formation^16^. Recent work has shown that this disinhibition mechanism is shared to varying degrees with other antagonist drugs with rapid-acting antidepressant efficacy, with ketamine being the most reliable^13^ and also demonstrating the most robust and sustained antidepressant effects^11^. In addition, there are a host of cascading intracellular changes following ketamine administration—involving eukaryotic elongation factor 2—that promote BDNF release^17,18^ and homeostatic synaptic scaling mechanisms^19^. Furthermore, cellular changes result from direct inhibition of extrasynaptic NMDA receptors^20^ that activate cellular plasticity mechanisms and promote synaptic potentiation. Given the evidence of direct and indirect changes in cortical excitation/inhibition (E/I) following ketamine administration, deriving noninvasive measures of E/I metrics in treatment-resistant MDD (TRD) participants could serve as useful biomarkers of antidepressant efficacy. Furthermore, E/I metrics have the potential to offer translational opportunities to fine-tune antidepressant response through adjunct pharmacological or neuromodulatory interventions.

We use dynamic causal modelling (DCM)^21^ to obtain non-invasively derived posterior E/I coupling parameters for a group of patients and controls under baseline, ketamine, and placebo scanning conditions. The groups were scanned with magnetoencephalography (MEG) during a passive somatosensory ‘airpuff’ stimulation task known to measure long-term potentiation and synaptic plasticity, important processes thought to mediate ketamine’s antidepressant effects^22^. DCM involves the fitting of parameterised mean-field models to electrophysiological data features in order to furnish parameter estimates of unobservable neuronal states. Previous findings suggest this approach offers the translational potential to characterise how ketamine alters receptor-mediated connectivity between various cell populations and modelled receptor dynamics^22^. This approach is supported by DCM literature—especially the work by Schmidt et al.^23^, which although focused on mismatch negativity, makes use of an established E/I (glutamate) biomarker to indicate the effects of ketamine on E/I balance in specific frontal pathways. We show that there is a relationship between the patients’ reported depression symptom improvement and the extent to which the ketamine infusion facilitates a shift toward the stable (top-left) quadrant of the Poincaré diagram. This validates the Poincaré diagram as a robust classification tool for TRD ketamine response. Furthermore, we show that this relationship does not arise by virtue of a placebo effect either in TRD patients or within the healthy control group. Finally, we show that both E and I coupling (rate constants) must increase in order for ketamine to shift patient neural dynamics in the desired direction in the Poincaré diagram. Future studies may be able to use our techniques to tailor the dosage of ketamine or to add adjunctive pharmacological or neuromodulatory treatments to shift a patient’s dynamics in any desired direction in the Poincaré diagram.

## Methods

### Data collection

All participants were studied as part of a larger double-blind, crossover clinical trial (NCT#00088699) of ketamine’s antidepressant efficacy at the National Institute of Mental Health in Bethesda MD. The present study included 18 participants (10 F/8 M, mean age = 36.9 ± 10.7 years) with a DSM-IV-TR diagnosis of MDD without psychotic features, who were experiencing a major depressive episode of at least 4 weeks duration and had a Montgomery Asberg Depression Rating Scale (MADRS)^24^ score of ≥20 at screening. They were also treatment-resistant, having failed at least one adequate antidepressant trial as assessed by the antidepressant treatment history form^25^. Healthy control participants (11 F/7 M, mean age = 33.9 ± 10.3 years) had no Axis I disorder or family history of Axis I disorder in first-degree relatives. The present study included only those participants who completed all baseline, post-ketamine and post-placebo scan sessions. Additional details on the sample and experimental design have been previously reported^26^.

Neuromagnetic data were collected at 1200 Hz (bandwidth 0–300 Hz) using a CTF 275-channel MEG system with SQUID-based axial gradiometers (VSM MedTech Ltd.) at baseline (i.e., not under the influence of any pharmaceutical agents), approximately 6–9 h following intravenous subanesthetic (0.5 mg/kg) ketamine infusion and approximately 6–9 h following intravenous placebo-saline infusion. Synthetic third gradient balancing was used to attenuate background environmental magnetic noise. Data were collected during a passive somatosensory stimulation task where participants received tactile stimulation of the right index finger (500 stimuli, 25-ms duration, 2-Hz average rate) over a 250-s experimental run. Tactile stimulation was controlled by a pneumatic stimulating device emitting brief bursts of air at 30 psi, displacing a plastic membrane placed against the skin of the distal phalange. During the experimental run, participants were instructed to focus on a stationary fixation dot projected on a screen in front of them. We administered the MADRS at multiple time points to measure the change in depression symptomatology for each condition. For baseline scores, we used the ratings collected 60 min prior to the first infusion. For ketamine and placebo scores, we used the ratings collected 230 min after each infusion. This time point reflects the closest rating collected relative to the MEG recording session. Change in MADRS scores was calculated by subtracting the post-ketamine and post-placebo ratings from the baseline rating—positive scores, therefore, reflect antidepressant efficacy.

### Data preprocessing

Offline, MEG data were visually inspected and trials were removed where visible artefacts including head movements, jaw clenches, eye blinks, and muscle movements were present. In addition, individual channels with excessive sensor noise were removed from subsequent analyses. Data were then bandpass filtered from 1 to 58 Hz and epoched from −100 to 300 ms peristimulus time using analysis routines available in the academic freeware SPM12 (Wellcome Trust Centre for Neuroimaging, http://www.fil.ion.ucl.ac.uk/spm/). Data for each participant and session were coregistered to a canonical template brain and source activity was extracted from the left primary somatosensory cortex (−40, −32, 60) with a 5 mm radius using SPM’s source-extraction algorithm. This algorithm extracts the principle eigenvariate, which like the average, is a summary of responses within a region of interest. Previous findings have demonstrated that these ‘virtual electrodes’ can suppress known artefacts in MEG data^27^. Subsequent DCM analysis used this wide-band, 1–58 Hz, virtual electrode signal. Filtering was applied to suppress environmental and physiological noise outside of these band limits^28^. For computational efficiency, we used the participant with the least number of trials remaining after data cleaning as the benchmark for subsequent analyses. Thus, we considered the first 389 trials for each participant and session in subsequent analyses. For each recording session, we used the first 121 time points (corresponding to 100 ms) following airpuff stimulation in the DCM analysis. We averaged these segments across all trials for each participant and session, to improve signal-to-noise by attenuating background noise, in order to obtain mean event-related potential (ERP) timecourses for each patient and control for each of the three scanning sessions.

### Bayesian model inversion

We use DCM to describe noninvasively-measured neural dynamics for patients with TRD and healthy control participants in terms of a coupled E/I model (see Appendix I). Participants were scanned during a somatosensory stimulation task, and neuromagnetic activity from the primary somatosensory cortex was utilised to examine neural dynamics for each subject and condition. Using the posterior estimates of the DCMs, we were then able to plot the coordinates summarising each subject’s dynamics under each scanning condition within the Poincaré diagram, as defined in dynamical systems theory (see Appendix II).

**Fig. 1:**
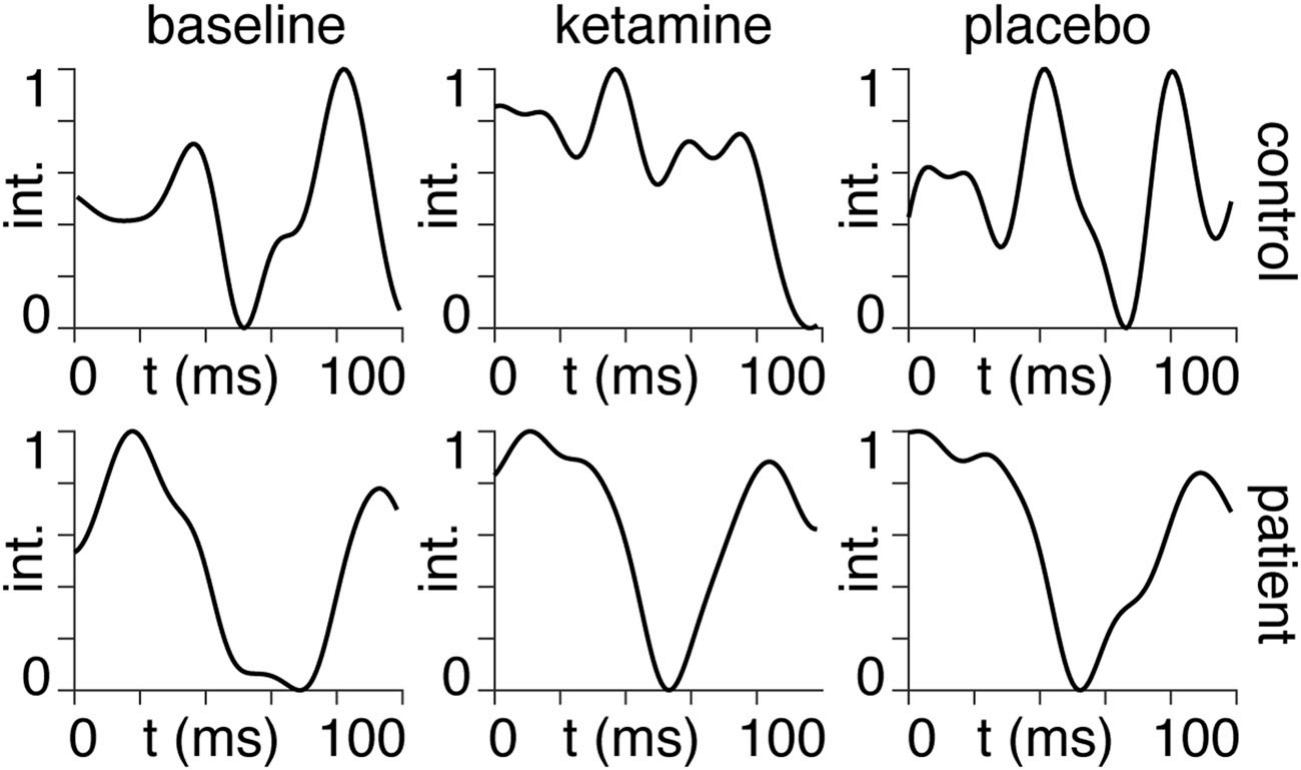
ERPs. Averaged ERP timecourses for the first 100 ms following airpuff stimulation (first-time point). The *y*-axis shows the MEG signal intensity (int.), normalised between zero and unity. The three conditions: baseline, ketamine and placebo are shown from left to right for a healthy control subject (top row) and a single TRD patient (bottom row).

We performed Bayesian model inversion using the spm_LAP inversion scheme in the SPM software. We use Eq. (2) (see Appendix I) as the flow function (equation of motion), together with an extrinsic input to each dependent variable accounting for the influence of the airpuff stimulation and an observer equation consisting of the sum of the E and I dependent variables (see accompanying code). We performed Bayesian model inversion on each of the 108-time series (18 subjects × 2 groups × 3 conditions), thereby obtaining posterior estimates for the E and I coupling strengths, from which we calculate a set of coordinates in the Poincaré diagram (see Appendix II). Bayesian model inversion was performed on ERP timecourses after averaging across all available trials for each subject. We show an example of these averaged timecourses for one patient and one healthy control under the three scanning sessions in Fig. 1. We show mean ERPs for the remaining 34 subjects in Supplementary Fig. 1.

### E/I and the Poincaré diagram

**Fig. 2:**
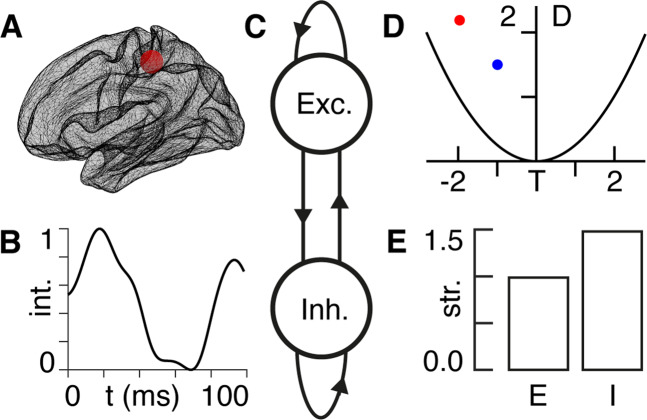
Methodology overview. **A** We obtain measurements of neural activity in the primary somatosensory cortex—approximate location shown by the red region. **B** Using non-invasive neuroimaging techniques, we obtain a timecourse of neural signal intensity (int.) from the primary somatosensory region in (**A**) following an airpuff stimulus—shown here for the TRD patient in baseline condition in Fig. 1. **C** We model the region of interest in (**A**) in terms of a coupled E/I model (see Appendix I), where the excitatory (Exc.) and inhibitory (Inh.) component are connected—both to themselves, as well as to each other—as indicated by the arrows. **D** The strengths of the E/I connections in (**C**) allow us to summarise the dynamical behaviour of the timecourse in (**B**) being expressed by the region in (**A**) by plotting its location (blue dot) within the Poincaré diagram (see Appendix II) with the trace (T) on the *x*-axis and the determinant (D) on the *y*-axis. The red dot indicates a known location with the Poincaré diagram that we would like to shift toward by a change in E/I. **E** This bar chart shows the relative amounts by which we need to vary the strengths (str.) of the E/I signals in (**C**) in order to shift the dynamics from the blue (current) to the red (target) dot in the Poincaré diagram in (**D**). For this example, E does not vary (strength = 1) and I needs to increase by 50% (see Appendix III).

We present a high-level overview of the methodology used in this paper in Fig. 2. Let us consider a neural region (Fig. 2A) in which the dynamics (Fig. 2B) are described by a balance between E and I (Fig. 2C, see Appendix I). The Poincaré diagram (Fig. 2D) then allows for an intuitive tool for the visualisation of system dynamics (see Appendix II). We derive the ways in which the E and I coupling strengths must be fine-tuned in order to achieve any desired shift in the Poincaré diagram (Fig. 2E). Although this technique allows for arbitrary shifts, going forward we focus on shifts ‘south-west’—as these turn out to be associated with symptom improvement (see “Results”). We show more examples of the relationship between E/I tuning parameters and source-target locations (Fig. 2D, E) in the Poincaré diagram in Supplementary Movie I.

## Results

### MADRS scores

We include mean MADRS scores for all 18 patients and controls under baseline, ketamine and placebo scanning conditions in Table 1. A full list of MADRS scores for all 18 participants and controls are provided in Supplementary Table 1.

**Table 1:** Mean and 95% confidence intervals for depression severity as measured by the MADRS, and posterior E/I coupling parameters for self-excitatory (A_EE), cross_excitatory (A_IE), cross-inhibitory (A_EI) and self-inhibitory (A_II) parameters obtained from Bayesian model inversion (see Appendix I).

	PATIENT			CONTROL		
	Baseline	Ketamine	Placebo	Baseline	Ketamine	Placebo
MADRS	33 [30.5–35.5]	27.5 [22.7–32.3]	31.4 [28.9–34.0]	1.8 [1.0–2.7]	1.8 [0.8–2.7]	1.1 [0.5 1.6]
A_EE	8.1 [2.2–14.1]	10.5 [6.2–14.9]	14.9 [10.2–19.7]	8.2 [4.5–11.9]	8.1 [0.6–15.6]	6.2 [−2.3 to 14.8]
A_IE	8.3 [2.4–14.2]	10.8 [6.5–15.1]	15.2 [10.3–20.0]	8.8 [5.2–12.5]	8.3 [0.8–15.7]	6.8 [−1.7 to 15.2]
A_EI	−8.4 [−14.3 to 2.5]	−10.9 [−15.2 to 6.6]	−15.2 [−20.0 to 10.5]	−8.5 [−12.1 to 4.8]	−8.3 [−15.8 to 0.9]	−6.6 [−15.1 to 1.9]
A_II	−8.1 [−14.0 to 2.1]	−10.5 [−14.8 to 6.2]	−14.9 [−20.0 to 10.1]	−8.1 [−11.8 to 4.4]	−8.1 [−15.6 to 0.5]	−6.2 [−14.7 to 2.4]

### Empirical E/I coupling parameters

We collect all the posterior E/I coupling parameters recovered by the Bayesian model inversion for patients and controls in the baseline, ketamine, and placebo conditions (see Table 1 and Supplementary Table 2). We now make some observations regarding the values in Supplementary Table 2. To begin with, we note that all four coupling parameters were given priors of zero and not constrained with regard to the sign (±) that they could adopt during the subsequent Bayesian model inversion (see accompanying code). Despite this freedom, with the exception of the fifth control subject in the ketamine condition, all cross-excitatory coupling parameters (A_IE) are positive and all cross-inhibitory coupling parameters (A_EI) are negative, which means that these models are composed of an excitatory and inhibitory component, as per the definitions in Appendix I. The fifth control subject in the ketamine condition has both A_IE < 0 and A_EI < 0, meaning that the best model describing this dataset is one composed of two coupled inhibitory regions, as opposed to a coupled excitatory-inhibitory region as with all others. Disregarding the fifth control subject in the ketamine condition, we note that all but two of the models (control #15 placebo and patient #1 placebo) are self-excitatory A_EE > 0 and self-inhibitory A_II < 0, rendering virtually all models as being composed of a fully-connected E-I system (see Appendix I).

### Plotting in the Poincaré diagram

**Fig. 3:**
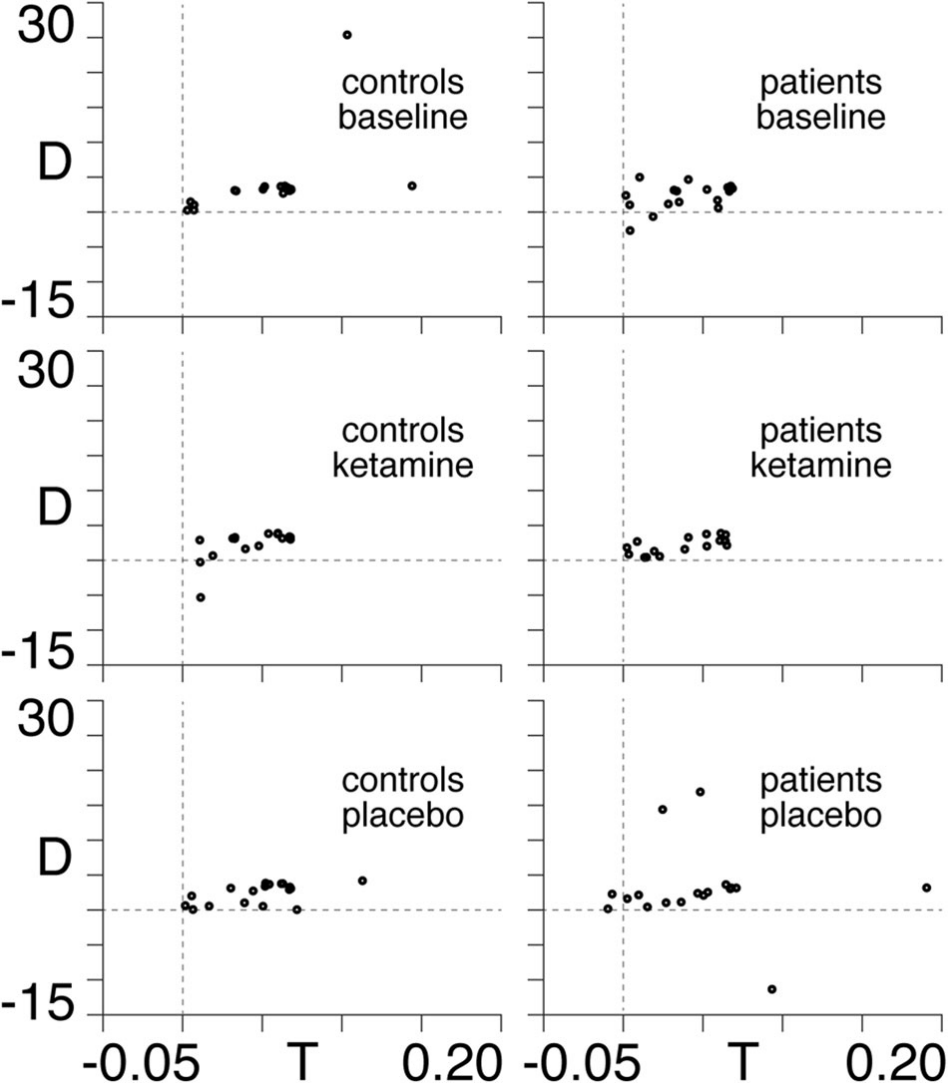
Results in the Poincaré diagram. Locations within the Poincaré diagram plotted for all controls (left column) and patients (right column) under baseline (first row), ketamine (middle row) and placebo (last row) conditions. The faint dotted lines show the axes of the trace (T)–determinant (D) plane within the Poincaré diagram.

We now use the posteriors obtained in Supplementary Table 2 to plot all models in the space of the Poincaré diagram (see Appendix II) in Fig. 3. We note from the top-right subplot in Fig. 3 that 16 of the 18 patients in the baseline condition have neural dynamics located in the top-right (unstable) quadrant of the Poincaré diagram.

### MADRS scores and the Poincaré diagram

**Fig. 4:**
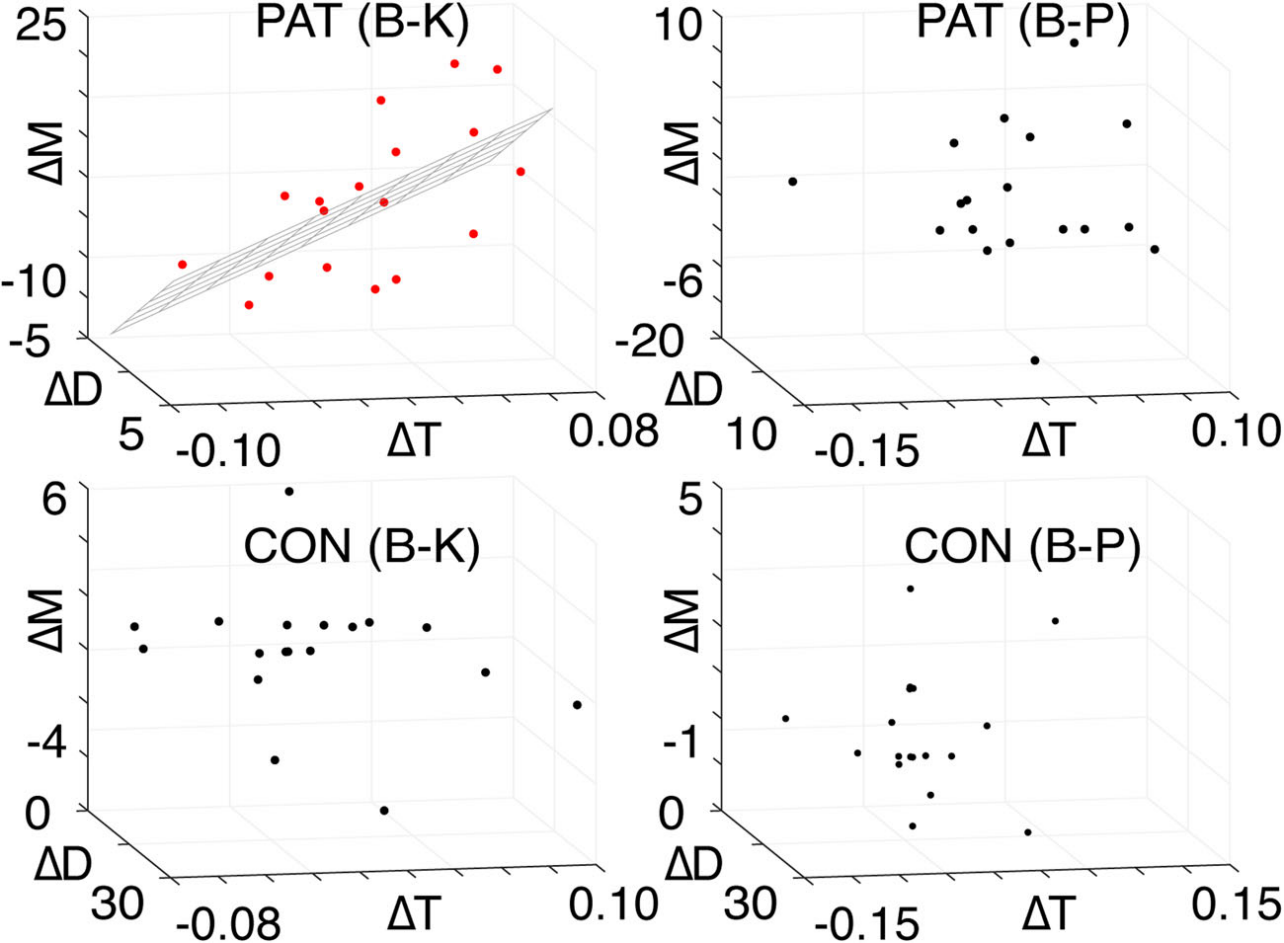
MADRS scores and the Poincaré diagram. The changes in the trace (∆*T*) and determinant (∆*D*) from baseline to ketamine (B-K, first column) and baseline to placebo (B-P, second column) for patients (PAT, first row) and controls (CON, second row), as a function of the associated change in MADRS (∆*M*) score. The hyperplane of the multiple linear regression is shown for the only significant model, PAT (B-K), with data points in red.

In what follows, we perform multiple linear regressions of the changes in MADRS scores (relative to baseline) by virtue of ketamine (B-K in Supplementary Table 1) and placebo (B-P in Supplementary Table 1) infusions against associated shifts within the Poincaré diagrams (Fig. 4). Note that this multiple linear regression technique allows us to treat the three-dimensional nature of the data (trace, determinant, and MADRS) within a single model^29^.

We find that a significant relationship (*p* = 0.01, *R*^2^ = 0.43) is observed for the baseline-to-ketamine conditions in the patient group, with regression coefficients: *β*_TR_ = 125.1 and *β*_DET_ = 1.7. In other words, we find that symptom improvement is associated with a southwest shift (trace and determinant decrease) in the Poincaré diagram (see Appendix III), with a dominant (99%) westerly component. This, together with the fact that 16 of the 18 patients begin in the northwest quadrant in the baseline condition (see Fig. 3), demonstrates that symptom improvement is associated with a shift from unstable dynamics (northeast quadrant) to stable dynamics (northwest quadrant) in the Poincaré diagram (see Appendix II). We find that the results remain when the two patients that do not begin in the northwest quadrant are excluded from the regression (*p* = 0.04, *R*^2^ = 0.39, *β*_TR_ = 118.2, *β*_DET_ = 2.4). No relationship is observed either in the baseline-placebo condition for the patient group, or for either condition in the control group. Furthermore, upon performing another multiple linear regression of the changes in each of the four individual coupling parameters (see Supplementary Table 2) between baseline and ketamine conditions in the patient group, we find that this model produces an *F* statistic of 3.7, as compared with the value of 6.2 obtained for the original trace and determinant model in Fig. 4. Therefore, the shifts in trace and determinant provide a better model of the changes in MADRS scores from baseline to ketamine in the patient group than their constituent E/I coupling parameters. This illustrates that the Poincaré diagram representation has explanatory power beyond the individual components of which the trace and determinant are comprised.

### Fine-tuning E/I for optimised treatment response

Having established the explanatory power of the Poincaré diagram (Fig. 4) and its link with E/I balance (see Appendix III), we are now in a position to ask the central question of this paper, namely, how should one fine-tune E/I balance in order to optimise treatment response? To answer this, we use the results in Fig. 4—the more the trace and determinant are decreased in patients by virtue of ketamine, the more their symptoms improve. If we perform the associated calculations (see Appendix III), we see that in order for this condition to be satisfied, ketamine must act in a way as to increase both E and I relative to the baseline condition.

## Discussion

We demonstrated that circuit-level excitatory and inhibitory coupling strengths (see Appendix I) can be derived noninvasively from neuromagnetic data, potentially offering an important first step towards personalised, rapid-acting antidepressant treatment for TRD patients. Numerous studies have demonstrated alterations in cortical E/I in MDD^30–32^, suggesting that a noninvasive measure of E/I coupling could provide a crucial step in identifying targeted treatments for depression. In addition, antidepressant-dose ketamine has been shown to alter cortical E/I by both directly inhibiting extrasynaptic NMDA receptors^20^ and indirectly increasing pyramidal cell excitability, downstream of synaptic GABAergic disinhibition^12,13^. These direct and indirect changes in E/I serve to increase BDNF expression^8^, AMPA surface expression^9,10^, and neuroplasticity-related signalling pathways and synaptic potentiation—mechanisms posited to be foundational to ketamine’s antidepressant efficacy^11^.

We have also demonstrated that the Poincaré diagram (see Appendix II) acts as a robust classification tool for predicting the efficacy of ketamine in the treatment of TRD. We show that south-westerly (dominantly westerly) shifts in the Poincaré diagram by virtue of ketamine are associated with antidepressant efficacy. This relationship does not exist following placebo saline infusion, suggesting that the Poincaré diagram acts as a biomarker of ketamine antidepressant response. Furthermore, we hypothesise that the mathematical relationships between E/I coupling strengths and associated shifts in the Poincaré diagram (see Appendix III) allow for the potential to individualise adjunctive pharmacological or neuromodulatory antidepressant interventions with ketamine. This relationship could, for example, leverage the downregulation of inhibition through the administration of a GABAergic inhibitor or the upregulation of excitation through repetitive paired-pulse transcranial magnetic stimulation—or even potentially through the titration of drug dosages for the maximisation of antidepressant efficacy. Briefly, this involves knowing a target direction in the Poincaré diagram, taken here to be a south-westerly shift from baseline condition (see Fig. 4). The beta values in the multiple linear regression indicate a strong (99%) preference for ‘west’ as the dominant direction. This, together with the fact that the patients in baseline condition are situated in the north-east quadrant of the Poincaré diagram (see Fig. 3), means that symptom improvement is associated with the extent of the shift toward the north-west quadrant. This in turn tells us that depressive symptom improvement is associated with the extent to which dynamics are shifted from unstable (north-east quadrant) toward stable (north-west quadrant) dynamics (see Appendix II). Note that the term ‘stability’ is here used in the context of dynamical systems theory—i.e. a system is said to be stable if it returns to equilibrium following a perturbative influence.

Once a target direction in the Poincaré diagram is known, we can calculate the precise amounts by which E/I coupling parameters need to be fine-tuned in order to optimise the effect of ketamine. We find that optimised shifts within the Poincaré diagram require an increase in both E and I coupling strengths (see Appendix III). It should be noted that these coupling strengths refer to rates of change in both E and I —rather than to E and I themselves. That is to say, an increase in E and I coupling strengths means that we require that the rates at which E and I increase (following a perturbation) must themselves increase. Increased cortical excitability has been previously reported to be associated with antidepressant response to ketamine in a time window overlapping with our measurement window in TRD patients^33^. As drug-induced increases in glutamate levels have been shown to return to baseline within two hours of ketamine administration^14^, this lingering increase in cortical excitability is thought to reflect enhanced AMPA receptor glutamatergic neurotransmission^33,34^. It is interesting to note that increases in inhibitory coupling are also associated with antidepressant response in our sample. Previous studies have demonstrated reduced GABA levels measured by magnetic resonance spectroscopy (MRS) in TRD patients^35,36^. In addition, animal work has demonstrated that sustained enhancement of GABAergic transmission is sufficient to elicit antidepressant-like behaviours^37^ and a pilot study of 11 MDD patients found that ketamine administration increased MRS-measured GABA concentrations up to 30 min after infusion^38^. Taken together, our findings suggest that changes in both excitatory drive (mediated by pyramidal cell disinhibition and subsequent AMPA throughput) and inhibitory drive (mediated by potentiation of GABAergic synaptic inhibition) are important for ketamine’s antidepressant efficacy in TRD patients.

While we have demonstrated a methodology to derive fine-tuning parameters for cortical E/I coupling in a TRD patient sample following ketamine administration and a group of healthy control subjects, several limitations should be noted. First, our reliance on manual methods for removing visible artefacts including head movements, jaw clenches, eye blinks and muscle movements can leave room for error in removing non-cortical sources of noise in the signal. However, we have attempted to control for this by extracting, filtering and averaging the principle eigenvariate from the primary somatosensory cortex to produce event-related fields of evoked activity from this cortical region. Second, our small TRD patient sample includes participants who have failed at least one antidepressant trial, making this sample relatively homogeneous. Our sample was also a convenience subsample of a larger clinical trial (i.e., participants completing all sessions of interest), which could have biased our results. Our methodology should be applied to a more heterogeneous and larger sample of depressed patients to determine how robust the derived metrics are for quantifying rapid-acting antidepressant response. Third, the Bayesian Model inversion assumes the most basic linear approximation of a coupled E/I system (see Appendix I), together with an equal balance of E and I contributing to measurement (see accompanying code). Future analyses on larger patient and control groups should be conducted with more complex models including biologically plausible parameters in order to assess trade-offs between complexity and accuracy. Fourth, several competing theories regarding ketamine’s rapid-acting mechanisms of action exist, as previously discussed. While our methods allow one to derive E/I coupling metrics non-invasively, questions remain about the direct relationship between macroscopic changes in E/I coupling and ketamine’s cellular and molecular processes and antidepressant mechanisms of action. Finally, the precise extents to which E and I should be increased depends upon the location in the Poincaré diagram towards which neural dynamics should be shifted. In other words, in order to be more specific than ‘both E and I should increase’, we must identify target coordinates in the Poincaré diagram (see Fig. 2 and Supplementary Movie I). This target could be taken, for example, as the average of a very large cohort of healthy controls in a baseline condition, or a similarly large group of patients that perform particularly well to treatment. Despite these limitations, we have proposed a simple, robust metric for deriving E/I coupling parameters non-invasively on a patient-by-patient basis that shows promise as a potential biomarker of antidepressant efficacy in TRD. Furthermore, we provide a link between neural dynamics, as quantified by coordinates in the Poincaré diagram, and tailored fine-tuning of E/I coupling strengths. We propose that the theoretical foundation, together with the outlined methodology presented here could be used in future clinical trials in order to move toward personalised medicine in the treatment of TRD.

## Appendix I

### Excitation/inhibition

Let us suppose that the dynamics in a given neural region can be described in terms of two time-dependent excitatory and inhibitory variables *E* and *I* which obey the following two coupled differential equations

1 $$\begin{array}{*{20}{c}} {\dot E = f\left( {E,I} \right)} \\ {\dot I = g\left( {E,I} \right)} \end{array}$$

where *f* and *g* are non-linear functions.

**Fig. 5:**
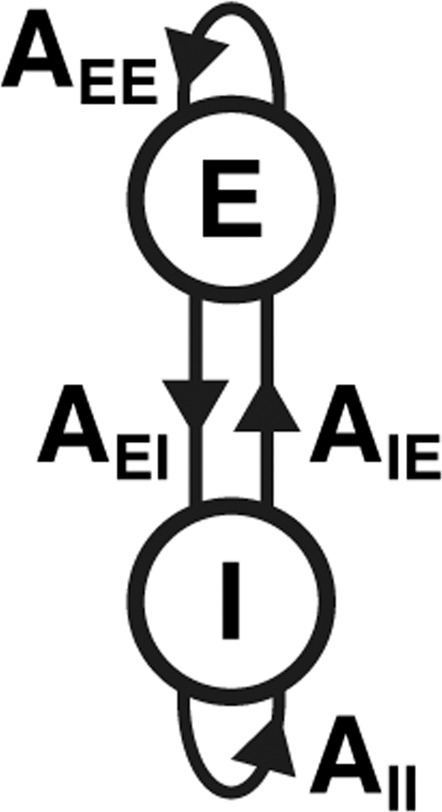
Excitatory/inhibitory coupling. An excitatory (E) and inhibitory (I) region, with E-to-E coupling (*A*_*EE*_), E-to-I coupling (*A*_*EI*_), I-to-E coupling (*A*_*IE*_) and I-to-I coupling (*A*_*II*_).

We can linearise the system in Eq (1) as follows:

2 $$\begin{array}{*{20}{c}} {\dot E = A_{EE}E + A_{EI}I} \\ {\dot I = A_{IE}E + A_{II}I} \end{array}$$

where $A_{EE} = \frac{{\partial \dot E}}{{\partial E}}$, $A_{EI} = \frac{{\partial \dot E}}{{\partial I}}$, $A_{IE} = \frac{{\partial \dot I}}{{\partial E}}$ and $A_{II} = \frac{{\partial \dot I}}{{\partial I}}$ are coupling parameters (see Fig. 5).

The constraints on the signs (whether positive or negative) of the coupling parameters in Eq. (2) depend on the specific context of the E/I system under consideration. For instance, in the predator-prey model described by the Lotka–Volterra equations^39^, both the self-excitatory (*A*_*EE*_) and cross-excitatory (*A*_*IE*_) coupling parameters must be positive and the self-inhibitory (*A*_*II*_) and cross-inhibitory (*A*_*EI*_) coupling parameters must be negative. On the other hand, Turing—in his derivation of the conditions required for spatial inhomogeneities (now known as Turing patterns^40^) to emerge in the mixture of a chemical E/I system—stipulated that only the signs of the self-coupling constants be fixed, with *A*_*EE*_ > 0 and *A*_*II*_ < 0. Within the context of neural systems, the two coupled differential equations of the E/I Wilson–Cowan model^41^ each contain a decay term, meaning that both E and I decrease if left unperturbed. In other words, the self-coupling constants *A*_*EE*_ and *A*_*II*_ are both negative. For the purpose of all analyses presented here, we stipulate that a pair of variables can be labelled as E and I if their excitatory cross-coupling constant is positive (*A*_*IE*_ > 0) and their inhibitory cross-coupling constant is negative (*A*_*EI*_ < 0)—with the self-coupling parameters being allowed to take any sign. That is to say, within a local neural region, the presence of an inhibitory signal will always decrease the amount of excitation and an excitatory signal will always increase the amount of inhibition, but if left uncoupled from one another, E and I are able to both increase and decrease with time.

It is important to keep in mind that when we address ‘E/I’ balance or fine-tuning strengths, we are referring to the relative sizes of the coupling parameters (rate constants) in Eq (2). A useful analogy in understanding the E/I balance comes from the classic predator–prey model of a population of chickens and foxes. If put in the same enclosure, the foxes will eat the chickens—i.e. the foxes are an inhibitor with a negative cross-coupling constant—as they decrease the chicken population. On the other hand, the chickens supply food to the foxes—i.e. the chickens are an excitatory with a positive cross-coupling constant—as their presence increases the fox population. When we talk about fine-tuning E/I strengths we are referring to changes in these coupling constants—e.g., increasing inhibition means that we increase the rate at which chickens will be eaten by foxes.

## Appendix II

### The Poincaré diagram

**Fig. 6:**
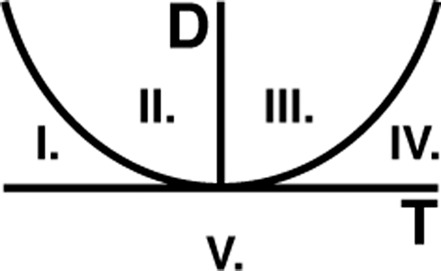
The Poincaré diagram. The Poincaré diagram, in which we plot the trace *T* against the determinant *D*, together with a curve defined by the quadratic: $D = \frac{1}{4}T^2$. We, therefore, divide the plane into the five labelled segments, each of which corresponds to a generic equilibrium: (I) Nodal sink; (II) Spiral sink; (III) Spiral source; (IV) Nodal source and (V) Saddle dynamics. Note that only the top-left quadrant (regions I and II) are stable—i.e., it is only in this region that dynamics will return to equilibrium following perturbation.

Eq. (2) in Appendix I can equivalently be written in matrix/vector notation as follows:

3 $$\left[ {\begin{array}{*{20}{c}} {\dot E} \\ {\dot I} \end{array}} \right] = \left[ {\begin{array}{*{20}{c}} {A_{EE}} & {A_{EI}} \\ {A_{IE}} & {A_{II}} \end{array}} \right]\left[ {\begin{array}{*{20}{c}} E \\ I \end{array}} \right]$$

the Jacobian *J* of which is given by

4 $$J = \left[ {\begin{array}{*{20}{c}} {A_{EE}} & {A_{EI}} \\ {A_{IE}} & {A_{II}} \end{array}} \right]$$

from which we obtain the trace *T* and determinant *D* defined as follows:

5 $$T = A_{EE} + A_{II}$$

6 $$D = A_{EE}A_{II} - A_{EI}A_{IE}$$

which we can plot against one another in order to succinctly represent the various dynamical regimes that can be displayed by the system in Eq. (2) (Fig. 6).

## Appendix III

### Excitation/inhibition and the Poincaré diagram

Let us examine the effect of fine-tuning the elements of the Jacobian in Eq. (4) as follows:

7 $$A_{EE} \to k_EA_{EE},\quad A_{IE} \to k_EA_{IE}$$

8 $$A_{II} \to k_IA_{II},\quad A_{EI} \to k_IA_{EI}$$

where *k*_*E*_ and *k*_*I*_ are positive constants—these are the fine-tuning parameters. Therefore, we assume that ketamine has the effect of fine-tuning the excitatory coupling parameters *A*_*EE*_ and *A*_*IE*_ by a constant *k*_*E*_ and the inhibitory coupling parameters *A*_*II*_ and *A*_*EI*_ by a constant *k*_*I*_. The fact that *k*_*E*_ and *k*_*I*_ are positive means that we are able to vary the magnitude (but not flip the signs) of the coupling parameters. In other words, we assume that the fine-tuning of pharmacological agents can result in a shift in E/I balance, but cannot reverse their roles by making E behave like I or vice versa.

We can then derive expressions for the new trace *T*_*k*_ and determinant *D*_*k*_, that result from applying the variations in Eqs. (7) and (8) to the original expressions in Eqs. (5) and (6)

9 $$T_k = k_EA_{EE} + k_IA_{II}$$

10 $$D_k = k_Ek_ID$$

Therefore, in order for the dynamics to shift southwest in the Poincaré diagram (see Appendix II) by virtue of the ketamine infusion, we require that *T*_*k*_ < *T* and *D*_*k*_ < *D* which, together with Eqs. (5), (6), (9), and (10), means that

11 $$k_EA_{EE} + k_IA_{II} < A_{EE} + A_{II}$$

12 $$k_Ek_ID < D$$

from which we obtain the following quadratic inequalities for *k*_*E*_ and *k*_*I*_

13 $$\frac{{A_{EE}}}{{A_{II}}}k_E^2 - \left( {\frac{{A_{EE}}}{{A_{II}}} + 1} \right)k_E + 1 < 0$$

14 $$\frac{{A_{II}}}{{A_{EE}}}k_I^2 - \left( {\frac{{A_{II}}}{{A_{EE}}} + 1} \right)k_I + 1 < 0$$

which have the following critical values

15 $$k_E = 1,\,k_E = \frac{{A_{II}}}{{A_{EE}}}$$

16 $$k_I = 1,\,k_I = \frac{{A_{EE}}}{{A_{II}}}$$

We are, therefore, now in a position to calculate the ranges of the E/I fine-tuning parameters *k*_*E*_ and *k*_*I*_ required to shift neural dynamics southwest in the Poincaré diagram.

We note from Eqs. (15) and (16) that the critical values depend only upon the self-coupling parameters *A*_*EE*_ and *A*_*II*_. Therefore, as long as *A*_*EE*_ and *A*_*II*_ have opposite ±signs, the fact that both *k*_*E*_ and *k*_*I*_ are restricted to positive values means that the inequalities in Eqs. (13) and (14) are only satisfied if both *k*_*E*_ > 1 and *k*_*I*_ > 1. In other words: both E and I must increase.

## Supplementary information

Supp. Figure 1

Supp. Figure 1 legend

Supp. Table 1

Supp. Table 2

Supp. Movie 1

Supp. Movie 1 legend

## Data Availability

All code is made available in the following public repository: https://github.com/allavailablepubliccode/TRD.
