# Supplementary figures and images for: Fine-tuning neural excitation/inhibition for tailored ketamine use in treatment-resistant depression
Source: Transl Psychiatry. 2021 May 29;11:335. doi: 10.1038/s41398-021-01442-3 (PMC8164631; doi:10.1038/s41398-021-01442-3)

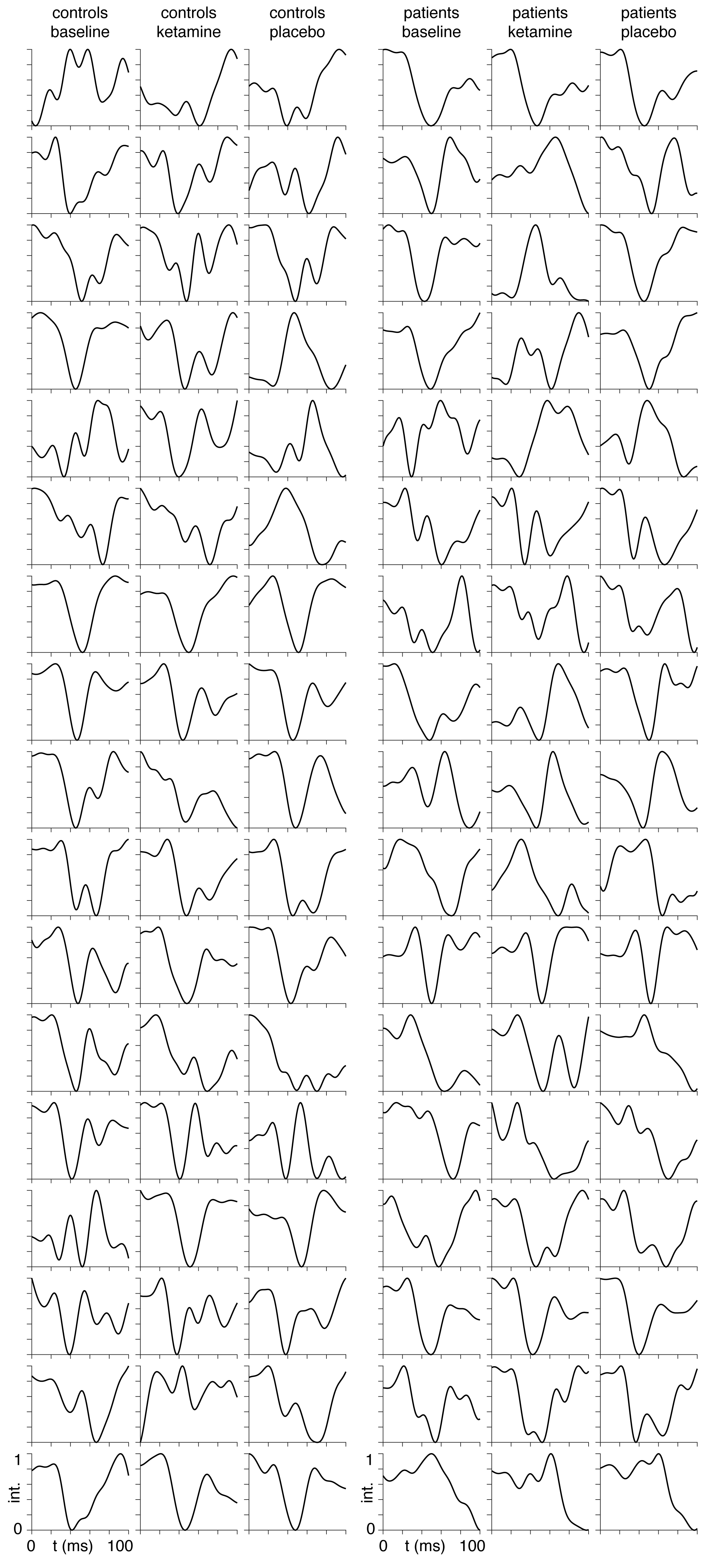

Supplement: Supplementary file 1 — Supp. Figure 1 [file 41398_2021_1442_MOESM1_ESM.tif]
