# Supplementary material for: Fine-tuning neural excitation/inhibition for tailored ketamine use in treatment-resistant depression
Source: Transl Psychiatry. 2021 May 29;11:335. doi: 10.1038/s41398-021-01442-3 (PMC8164631; doi:10.1038/s41398-021-01442-3)
Supplement: Supplementary file 2 — Supp. Figure 1 legend [file 41398_2021_1442_MOESM2_ESM.docx]

**Supplementary Figure 1: ERPs.** Averaged ERP timecourses for the first 100ms following airpuff stimulation (first time point). The y-axis shows the MEG signal intensity (int.), normalized between zero and unity. The three sessions: baseline, ketamine, and placebo are shown from left-to-right for all the controls (first three columns) and patients (last three columns), excluding the two subjects shown in Figure 1.
