# Supplementary material for: Fine-tuning neural excitation/inhibition for tailored ketamine use in treatment-resistant depression
Source: Transl Psychiatry. 2021 May 29;11:335. doi: 10.1038/s41398-021-01442-3 (PMC8164631; doi:10.1038/s41398-021-01442-3)
Supplement: Supplementary file 3 — Supp. Table 1 [file 41398_2021_1442_MOESM3_ESM.docx]

| **Sub** | **B** | **P** | **K** | **B-K** | **B-P** | **Sub** | **B** | **P** | **K** | **B-K** | **B-P** |
| --- | --- | --- | --- | --- | --- | --- | --- | --- | --- | --- | --- |
| **P01** | 31 | 32 | 24 | 7 | -1 | **H01** | 0 | 0 | 0 | 0 | 0 |
| **P02** | 41 | 35 | 31 | 10 | 6 | **H02** | 3 | 2 | 3 | 0 | 1 |
| **P03** | 38 | 33 | 20 | 18 | 5 | **H03** | 6 | 1 | 0 | 6 | 5 |
| **P04** | 25 | 25 | 28 | -3 | 0 | **H04** | 1 | 2 | 0 | 1 | -1 |
| **P05** | 31 | 29 | 37 | -6 | 2 | **H05** | 1 | 1 | 0 | 1 | 0 |
| **P06** | 35 | 35 | 11 | 24 | 0 | **H06** | 0 | 0 | 1 | -1 | 0 |
| **P07** | 26 | 24 | 25 | 1 | 2 | **H07** | 0 | 1 | 0 | 0 | -1 |
| **P08** | 33 | 24 | 9 | 24 | 9 | **H08** | 1 | 1 | 0 | 1 | 0 |
| **P09** | 36 | 36 | 41 | -5 | 0 | **H09** | 1 | 3 | 0 | 1 | -2 |
| **P10** | 30 | 38 | 36 | -6 | -8 | **H10** | 0 | 0 | 4 | -4 | 0 |
| **P11** | 30 | 30 | 35 | -5 | 0 | **H11** | 0 | 0 | 6 | -6 | 0 |
| **P12** | 24 | 21 | 10 | 14 | 3 | **H12** | 2 | 1 | 2 | 0 | 1 |
| **P13** | 38 | 35 | 43 | -5 | 3 | **H13** | 4 | 0 | 5 | -1 | 4 |
| **P14** | 28 | 29 | 25 | 3 | -1 | **H14** | 1 | 0 | 0 | 1 | 1 |
| **P15** | 32 | 32 | 25 | 7 | 0 | **H15** | 2 | 0 | 1 | 1 | 2 |
| **P16** | 35 | 29 | 24 | 11 | 6 | **H16** | 4 | 4 | 4 | 0 | 0 |
| **P17** | 40 | 41 | 35 | 5 | -1 | **H17** | 3 | 1 | 3 | 0 | 2 |
| **P18** | 41 | 38 | 36 | 5 | 3 | **H18** | 4 | 2 | 3 | 1 | 2 |

**Supplementary Table 1: Sub = subject; B = baseline; P = placebo; K = ketamine.** MADRS scores for all patients P01-P18 and controls H01-H18, including the changes in MADRS scores by virtue of ketamine (B-K) and placebo (B-P) infusions. Note that P01 and H01 correspond to the two subjects shown in Figure 1 and all remaining subjects correspond to those shown in Supplementary Figure 1, in the same order.
