# Supplementary material for: Fine-tuning neural excitation/inhibition for tailored ketamine use in treatment-resistant depression
Source: Transl Psychiatry. 2021 May 29;11:335. doi: 10.1038/s41398-021-01442-3 (PMC8164631; doi:10.1038/s41398-021-01442-3)
Supplement: Supplementary file 4 — Supp. Table 2 [file 41398_2021_1442_MOESM4_ESM.docx]

| **CONTROLS** | **BASELINE** | | | |  | **KETAMINE** | | | |  | **PLACEBO** | | | |
| --- | --- | --- | --- | --- | --- | --- | --- | --- | --- | --- | --- | --- | --- | --- |
|  | **A_EE** | **A_IE** | **A_EI** | **A_II** |  | **A_EE** | **A_IE** | **A_EI** | **A_II** |  | **A_EE** | **A_IE** | **A_EI** | **A_II** |
|  | 7.68 | 7.80 | -7.92 | -7.61 |  | 7.94 | 8.06 | -8.18 | -7.87 |  | 4.27 | 4.59 | -4.63 | -4.24 |
|  | 0.04 | 0.52 | -0.52 | -0.04 |  | 5.08 | 5.36 | -5.48 | -5.02 |  | 1.84 | 2.77 | -2.67 | -1.73 |
|  | 5.44 | 5.68 | -5.72 | -5.41 |  | 4.73 | 5.02 | -5.06 | -4.70 |  | 1.02 | 1.38 | -1.48 | -0.98 |
|  | 11.42 | 11.46 | -11.56 | -11.36 |  | 2.32 | 2.65 | -2.77 | -2.28 |  | 1.78 | 1.84 | -1.96 | -1.72 |
|  | 8.91 | 8.99 | -9.11 | -8.84 |  | 0.51 | -0.04 | -0.07 | -0.50 |  | 9.70 | 9.76 | -9.88 | -9.64 |
|  | 5.34 | 5.57 | -5.66 | -5.29 |  | 4.21 | 4.48 | -4.60 | -4.14 |  | 5.48 | 5.72 | -5.84 | -5.42 |
|  | 58.08 | 58.27 | -58.22 | -57.98 |  | 6.61 | 6.11 | -6.27 | -6.60 |  | 2.74 | 3.13 | -3.24 | -2.70 |
|  | 7.74 | 7.86 | -7.98 | -7.68 |  | 7.69 | 7.81 | -7.93 | -7.62 |  | 5.20 | 5.47 | -5.57 | -5.15 |
|  | 8.26 | 8.36 | -8.48 | -8.20 |  | 2.90 | 3.37 | -3.44 | -2.87 |  | 9.17 | 9.25 | -9.36 | -9.10 |
|  | 5.10 | 5.36 | -5.48 | -5.04 |  | 8.68 | 8.77 | -8.89 | -8.62 |  | 5.86 | 6.09 | -6.21 | -5.80 |
|  | 3.70 | 4.09 | -4.20 | -3.65 |  | 2.48 | 3.00 | -3.01 | -2.47 |  | 0.86 | 0.79 | -0.94 | -0.79 |
|  | 6.64 | 6.83 | -6.87 | -6.61 |  | 5.44 | 5.68 | -5.72 | -5.40 |  | 3.39 | 3.78 | -3.89 | -3.34 |
|  | 3.59 | 3.99 | -4.05 | -3.45 |  | 4.93 | 5.21 | -5.33 | -4.87 |  | 45.78 | 45.77 | -45.79 | -45.76 |
|  | 6.72 | 6.89 | -7.01 | -6.66 |  | 20.94 | 20.92 | -20.99 | -20.90 |  | 0.31 | 0.86 | -0.86 | -0.31 |
|  | 0.55 | 0.77 | -0.77 | -0.54 |  | 5.40 | 5.64 | -5.69 | -5.36 |  | -0.13 | 0.31 | -0.31 | 0.13 |
|  | 0.21 | 1.03 | -1.06 | -0.20 |  | 41.09 | 41.08 | -41.11 | -41.07 |  | 4.93 | 5.25 | -5.32 | -4.88 |
|  | 6.22 | 6.42 | -6.54 | -6.15 |  | 4.97 | 5.27 | -5.38 | -4.92 |  | 8.02 | 8.13 | -8.25 | -7.95 |
|  | 1.06 | 1.61 | -1.61 | -1.05 |  | 10.06 | 10.12 | -10.23 | -9.99 |  | 1.70 | 2.21 | -2.22 | -1.69 |
|  |  |  |  |  |  |  |  |  |  |  |  |  |  |  |
| **PATIENTS** | 4.91 | 5.18 | -5.30 | -4.85 |  | 5.39 | 5.65 | -5.77 | -5.33 |  | -0.07 | 0.42 | -0.44 | 0.06 |
|  | 1.63 | 1.98 | -2.16 | -1.57 |  | 2.99 | 3.43 | -3.52 | -2.95 |  | 8.80 | 8.89 | -9.00 | -8.73 |
|  | 9.48 | 9.54 | -9.66 | -9.41 |  | 4.62 | 4.83 | -4.95 | -4.56 |  | 6.41 | 6.60 | -6.72 | -6.35 |
|  | 0.65 | 1.20 | -1.21 | -0.64 |  | 0.41 | 1.22 | -1.21 | -0.39 |  | 1.11 | 1.49 | -1.57 | -1.07 |
|  | 2.99 | 3.71 | -3.74 | -2.98 |  | 6.33 | 6.53 | -6.65 | -6.27 |  | 15.78 | 15.78 | -15.87 | -15.73 |
|  | 6.77 | 6.95 | -6.99 | -6.73 |  | 0.62 | 1.11 | -1.11 | -0.61 |  | 0.39 | 1.32 | -1.34 | -0.39 |
|  | 0.92 | 1.11 | -1.26 | -0.86 |  | 51.86 | 51.85 | -51.87 | -51.85 |  | 8.93 | 9.01 | -9.13 | -8.86 |
|  | 5.66 | 5.89 | -6.02 | -5.60 |  | 0.45 | 0.84 | -0.91 | -0.43 |  | 56.52 | 56.63 | -56.64 | -56.49 |
|  | 6.82 | 6.98 | -7.11 | -6.76 |  | 10.74 | 10.78 | -10.89 | -10.68 |  | 2.77 | 3.11 | -3.21 | -2.73 |
|  | 5.30 | 5.03 | -5.06 | -5.30 |  | 4.14 | 4.30 | -4.42 | -4.07 |  | 3.14 | 3.44 | -3.53 | -3.14 |
|  | 8.25 | 8.35 | -8.47 | -8.18 |  | 4.56 | 4.88 | -4.99 | -4.51 |  | 1.12 | 1.82 | -1.87 | -1.11 |
|  | 5.83 | 6.04 | -6.12 | -5.78 |  | 1.95 | 2.53 | -2.55 | -1.94 |  | 1.98 | 2.64 | -2.55 | -1.79 |
|  | 28.70 | 28.69 | -28.73 | -28.68 |  | 11.06 | 11.10 | -11.21 | -11.00 |  | 45.51 | 45.65 | -45.69 | -45.46 |
|  | 1.20 | 0.87 | -0.88 | -1.18 |  | 0.77 | 1.53 | -1.57 | -0.77 |  | 30.16 | 30.14 | -30.19 | -30.13 |
|  | 21.04 | 21.09 | -21.09 | -21.03 |  | 21.17 | 21.16 | -21.22 | -21.13 |  | 2.72 | 3.07 | -3.19 | -2.66 |
|  | 7.87 | 7.99 | -8.10 | -7.80 |  | 54.31 | 54.30 | -54.32 | -54.30 |  | 50.25 | 50.23 | -50.25 | -50.23 |
|  | 4.81 | 5.23 | -5.28 | -4.77 |  | 5.37 | 5.63 | -5.75 | -5.31 |  | 9.18 | 9.25 | -9.37 | -9.11 |
|  | 23.20 | 23.18 | -23.24 | -23.17 |  | 3.01 | 3.25 | -3.37 | -2.96 |  | 23.93 | 23.63 | -23.66 | -23.83 |

**Supplementary Table 2:** Self-excitatory (A_EE), cross-excitatory (A_IE), cross-inhibitory (A_EI), and self-inhibitory (A_II) coupling parameters (see Appendix I) obtained from the posteriors of the Bayesian model inversion of all 18 controls and patients under baseline, ketamine, and placebo conditions.
