# Supplementary material for: Fine-tuning neural excitation/inhibition for tailored ketamine use in treatment-resistant depression
Source: Transl Psychiatry. 2021 May 29;11:335. doi: 10.1038/s41398-021-01442-3 (PMC8164631; doi:10.1038/s41398-021-01442-3)
Supplement: Supplementary file 6 — Supp. Movie 1 legend [file 41398_2021_1442_MOESM6_ESM.docx]

**Supplementary Movie I: E/I balance and the Poincaré diagram.** Left: An initial pre-ketamine (blue dot) location and a final post-ketamine (red dot) location in the Poincaré diagram with the Trace (T) on the x-axis and Determinant (D) on the y-axis (see Appendix II). Right: The excitation (E) and inhibition (I) fine-tuning parameters necessary to achieve the shift from the blue to the red dot in the Poincaré diagram on the left.
